# Discovering cell types using manifold learning and enhanced visualization of single-cell RNA-Seq data

**DOI:** 10.1038/s41598-021-03613-0

**Published:** 2022-01-07

**Authors:** Akram Vasighizaker, Saiteja Danda, Luis Rueda

**Affiliations:** School of Computer Science, University of Windsor, Windsor, ON Canada

**Keywords:** Computer science, Computational models, Machine learning

## Abstract

Identifying relevant disease modules such as target cell types is a significant step for studying diseases. High-throughput single-cell RNA-Seq (scRNA-seq) technologies have advanced in recent years, enabling researchers to investigate cells individually and understand their biological mechanisms. Computational techniques such as clustering, are the most suitable approach in scRNA-seq data analysis when the cell types have not been well-characterized. These techniques can be used to identify a group of genes that belong to a specific cell type based on their similar gene expression patterns. However, due to the sparsity and high-dimensionality of scRNA-seq data, classical clustering methods are not efficient. Therefore, the use of non-linear dimensionality reduction techniques to improve clustering results is crucial. We introduce a method that is used to identify representative clusters of different cell types by combining non-linear dimensionality reduction techniques and clustering algorithms. We assess the impact of different dimensionality reduction techniques combined with the clustering of thirteen publicly available scRNA-seq datasets of different tissues, sizes, and technologies. We further performed gene set enrichment analysis to evaluate the proposed method’s performance. As such, our results show that modified locally linear embedding combined with independent component analysis yields overall the best performance relative to the existing unsupervised methods across different datasets.

## Introduction

Single-cell sequencing is an emerging technology used to capture cell information at a single-nucleotide resolution and by which individual cells can be analyzed separately^1^. As of now, single-cell RNA-seq (scRNA-seq) datasets have been generated for different purposes^2^. However, these high-dimensional and sparse data lead to some analytical challenges. While many computational methods have been successfully proposed for analyzing scRNA-seq data, there are still some open problems in this research area. One of the main challenges is the sparsity of the data and the curse of dimensionality present in scRNA-seq data. Also, performing well-defined pre-processing steps leads to enhancing the quality of data and new biological insights. Analyzing scRNA-seq data can be divided into two main categories: cell and gene levels. Finding cell sub-types or highly differentially expressed tissue-specific gene set is one of the common challenges at the cell level^3^. Arranging cells into clusters to find the data’s heterogeneity is arguably the most significant step of any scRNA-seq data downstream analysis. This step could be used to distinguish tissue-specific sub-types based on identified gene sets. Indeed, cell clustering aims to identify cell types based on the patterns embedded in gene expression without prior knowledge at the cell level. Since the number of genes that are profiled in scRNA-seq data is typically large, cells tend to be located close to each other via non-metric distances, but rather complex relationships in high-dimensional spaces^4^. Therefore, traditional dimensionality reduction and clustering algorithms are not efficient for these scenarios, and hence, they cannot efficiently separate individual cell types. Several algorithms have been proposed to this aim and alleviate the problem of the curse of dimensionality.

Dimensionality reduction techniques have been widely used in large-scale scRNA-seq data processing^5^. Most of the previous studies use principal component analysis (PCA). However, one of the main drawbacks of PCA is that it cannot deal with sparse matrices and non-metric relationships among high-dimensional data points. Other works have also employed PCA as a pre-processing step to remove cell outliers for dimensionality reduction and visualization. Other methods proposed non-linear dimensionality reduction, including *t*-distributed Stochastic Neighborhood Embedding (*t*-SNE)^6^. This method is able to preserve the local structure of data, although it is not efficient applicable when applied on very large datasets^7^.

Moreover, various studies have used unsupervised clustering models to identify rare novel cell types. For instance, the hierarchical clustering algorithm divides large clusters into smaller ones or progressively merges each data point into larger clusters. This algorithm has been employed to analyze scRNA-seq data by BackSPIN^8^ and pcaReduce^9^, through dimensionality reduction after each division or combination in an iterative manner. *k*-means, which is one of the most common clustering algorithms, has been employed in the Monocle, specifically for analyzing scRNA-seq data^10^. Also, the authors of^11^ used the Louvain algorithm, which is based on community detection techniques to analyze complex networks^12^.

However, to achieve acceptable clustering performance on scRNA-seq data, other comprehensive studies indicated that hybrid models, designed as a combination of clustering and dimensionality reduction techniques, tend to improve the clustering results^13^. They learned 20 different models using four dimensionality reduction methods, including PCA, non-negative matrix factorization (NMF), filter-based feature selection (FBFS), and Independent Component Analysis (ICA). They also used five clustering algorithms as *k*-means, density-based spatial clustering with noise (DBSCAN), fuzzy *c*-means, Louvain, and hierarchical clustering. Their experiments highlighted the positive effect of hybrid models and showed that using feature-extraction methods could be a decent way to improve clustering performance. Their experimental results indicate that Louvain combined with ICA performed well in small feature spaces.

This paper proposes a model to obtain the well-separated and meaningful clusters of cells from large-scale scRNA-seq data. We focus on the combination of unsupervised dimensionality reduction followed by conventional clustering. We discovered a hybrid model of non-linear dimensionality reduction technique, MLLE, and linear combination method, ICA for visualization. We used PCA, t-SNE, Isomap, standard Locally Linear Embedding (LLE), and Laplacian eigenmaps to perform a comparative analysis on diverse methods. ICA is employed to enhance the visualization of clustered data. Parameter tuning or choosing the best parameters for dimensionality reduction and clustering has been one of the critical challenges, and that is well addressed in our work. Experimental results on thirteen different benchmark scRNA-seq datasets show the power of modified LLE and ICA on the representation quality of the clustering data, providing very high accuracy and enhanced visualization. Confirmatory biological annotations were observed in the clusters using corresponding marker genes found by our method.

## Materials and methods

**Figure 1:**
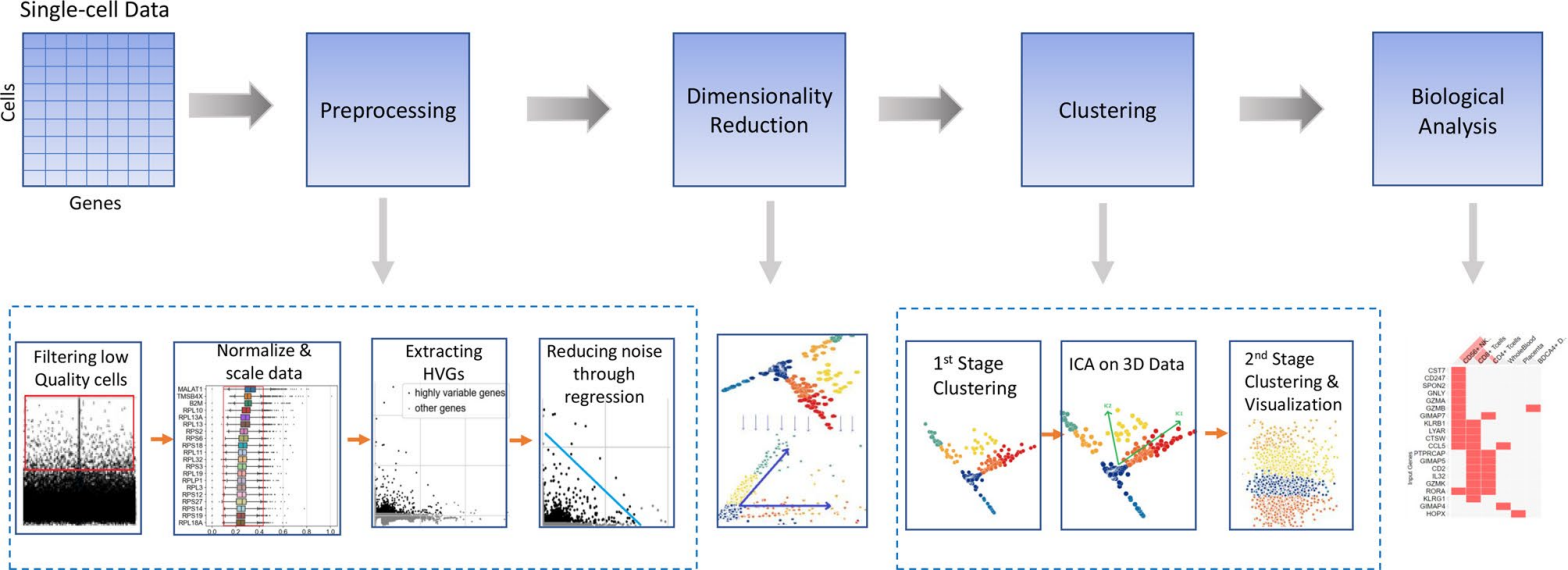
Block diagram of the proposed approach for discovering cell types in scRNA-seq data.

The block diagram of the proposed pipeline is depicted in Fig. 1. First, the scRNA-seq data is pre-processed based on the number of cells and the number of genes. Highly variable genes are extracted as part of the feature selection step after normalization and scaling of the filtered data. Linear regression is one of the most widely-used methods to regress out potential sources of variation presented in the data based on the total counts per cell and mitochondrial percentage as discussed in Refs.^11,14^. The data obtained at this point is then processed to reduce the feature space into two or three dimensions; afterward, *k*-means clustering is applied. In addition, we performed ICA on the lower-dimensional data followed by *k*-means clustering to achieve improved visualization and meaningful clusters.

### Datasets

To evaluate the performance of the proposed method, a total of thirteen benchmark datasets were used, which include single-cell gene expression profiles. The details of all datasets used in this work are given in Table 1. They vary across size, tissue (pancreas, lung, peripheral blood), sequencing protocol (three different protocols), and species (Human and Mouse). Peripheral blood dataset, 3k PBMC from a healthy donor, were downloaded from the 10$X$ Genomics portal^15^. Pancreas datasets include Baron (GSE84133)^16^ , Muraro (GSE85241)^17^, Segerstolpe (EMTAB-5061)^18^, Xin (GSE81608)^19^, and Wang^20^. In lung datasets, H1299 scRNA-seq (GSE148729)^21^ and Calu3 scRNA-seq (GSE148729)^21^, different cell lines were contaminated with SARS-CoV-1 and SARS-CoV-2 and sequenced at different time slots. These datasets are unlabeled and do not have any background knowledge of the data. In this case, we analyzed the data and provided useful information about the unknown data. On the other hand, the cell-type annotation for PBMC, Baron, Segerstolpe, and Wang was provided with the data. We used these annotations as “background knowledge” during the evaluation of the clustering method. To make a fair assessment of the clustering methods, the cell annotations were removed, and the clustering was done. Then, the labels were considered to compare the biological results.

**Table 1:** Datasets used in this work.

Dataset	No. of cells	No. of genes	Accession number	Description	Sequencing technology
Baron_human1	16,381	1937	GSE84133	Human pancreas	llumina HiSeq 2500 (inDrop)
Baron_human2	16,381	1724	GSE84133	Human pancreas	llumina HiSeq 2500 (inDrop)
Baron_human3	16,381	3605	GSE84133	Human pancreas	llumina HiSeq 2500 (inDrop)
Baron_human4	16,381	1303	GSE84133	Human pancreas	llumina HiSeq 2500 (inDrop)
Baron_mouse1	14,878	822	GSE84133	Mouse pancreas	llumina HiSeq 2500 (inDrop)
Baron_mouse2	14,878	1064	GSE84133	Mouse pancreas	llumina HiSeq 2500 (inDrop)
Muraro	17,140	3071	GSE85241	Human pancreas	Illumina NextSeq 500 (CEL-Seq2)
Segerstolpe	26,271	7028	E_MTAB_5061	Human pancreas	Smart-Seq2
Xin	39,851	1601	GSE81608	Human Pancreas	Illumina HiSeq 2500(SMARTer)
Wang	19,950	635	GSE83139	Human Pancreas	Illumina HiSeq 2000(SMARTer)
H1299 scRNA-seq	48,890	27,072	GSE148729	Human lung (SARS-CoV-2)	Illumina NextSeq 500
Calu3 scRNA-seq	24,754	27,072	GSE148729	Human lung (SARS-CoV-2)	Illumina NextSeq 500
PBMC	2700	32,738	10 $\times$ Genomics (pbmc3k)	3k PBMCs from a Healthy donor	Cell ranger

### Data pre-processing and quality control

A common practice for generating RNA-seq raw data is to use next-generation sequencing technologies to create read count matrices. The read count data matrix contains gene names and their expression levels across individual cells. Before analyzing scRNA-seq data, one needs to ensure that gene expressions and cells are of standard quality. We followed a typical scRNA-seq analysis workflow including quality control, as described in^14,22^. A Python package, Scanpy, is used to perform pre-processing and quality control steps. Based on the expression levels, we filtered out weakly expressed genes and low-quality cells in which fewer reads are mapped, as shown in Fig. 1, the first step of pre-processing. Low-quality cells that are dead, degraded, or damaged during sequencing are represented by a low number of expressed genes. Genes expressed in less than three cells and cells with less than 200 expressed genes are removed.

**Figure 2:**
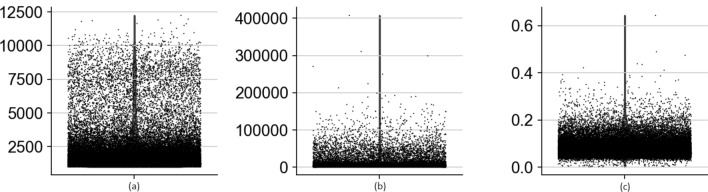
Investigating the distribution of the data to filtered out weakly expressed genes and low-quality cells from dataset; (**a**) number of expressed genes, (**b**) total counts per cell, and (**c**) the percentage of mitochondrial genes for H1299 scRNA-seq.

We also investigated the distribution of the data (Fig. 2) as a data-specific quality-control step and filtered out low-quality cells and genes. We removed cells with a high percentage of mitochondrial gene counts. Mitochondrial genes do not contribute significant information to the downstream analysis^22,23^. Also, some cells present large total counts compared with other cells, indicating potential sources of variation. To reduce this effect, we scaled the data to unit variance. Since scRNA-seq data are expressed at different levels, normalization is a must. Normalization is the method of translating numeric columns’ values in a dataset to a standard scale without distorting the ranges of values. We normalize the data using the Counts Per Million (CPM) normalization combined with logarithmic scaling on the data:

1 $$\begin{aligned} CPM= readsMappedToGene \times \frac{1}{totalReads} \times 10^6. \end{aligned}$$

where *totalReads* is the total number of mapped reads of a sample, and *readsMappedToGene* is the number of reads mapped to a selected gene.

**Figure 3:**
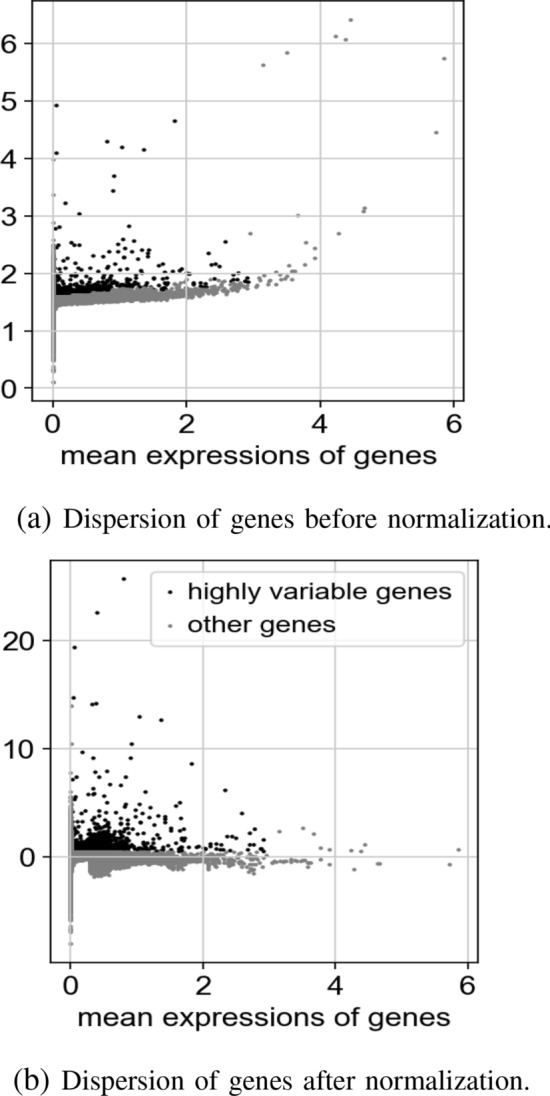
Comparison of dispersion of normalized and not normalized genes to extract highly variable genes.

At this point, we extracted highly variable genes (HVGs) as a part of the feature selection step, aiming at minimizing the search space, and only these genes are examined in further evaluation. HVGs are those genes that are expressed significantly more or less in some cells compared to other ones. This step in quality control makes sure that the differences occur because of biological differences and not technical noise. The simplest approach to compute such a variation is to quantify the variance of the expression values for each gene across all the samples. A good trade-off between mean and variance would help select the subset of genes that keep useful biological knowledge, while removing noise. We use log-normalized data because we want to ensure having the same log-values in the clustering and dimensionality reduction follow a consistent analysis through all steps. There are conventional approaches to find the best threshold. The normalized dispersion is obtained by scaling the mean and standard deviation of the dispersion for genes falling into a given bin for the mean of expressions (Fig. 3). This means that HVGs are selected for each bin.

**Figure 4:**
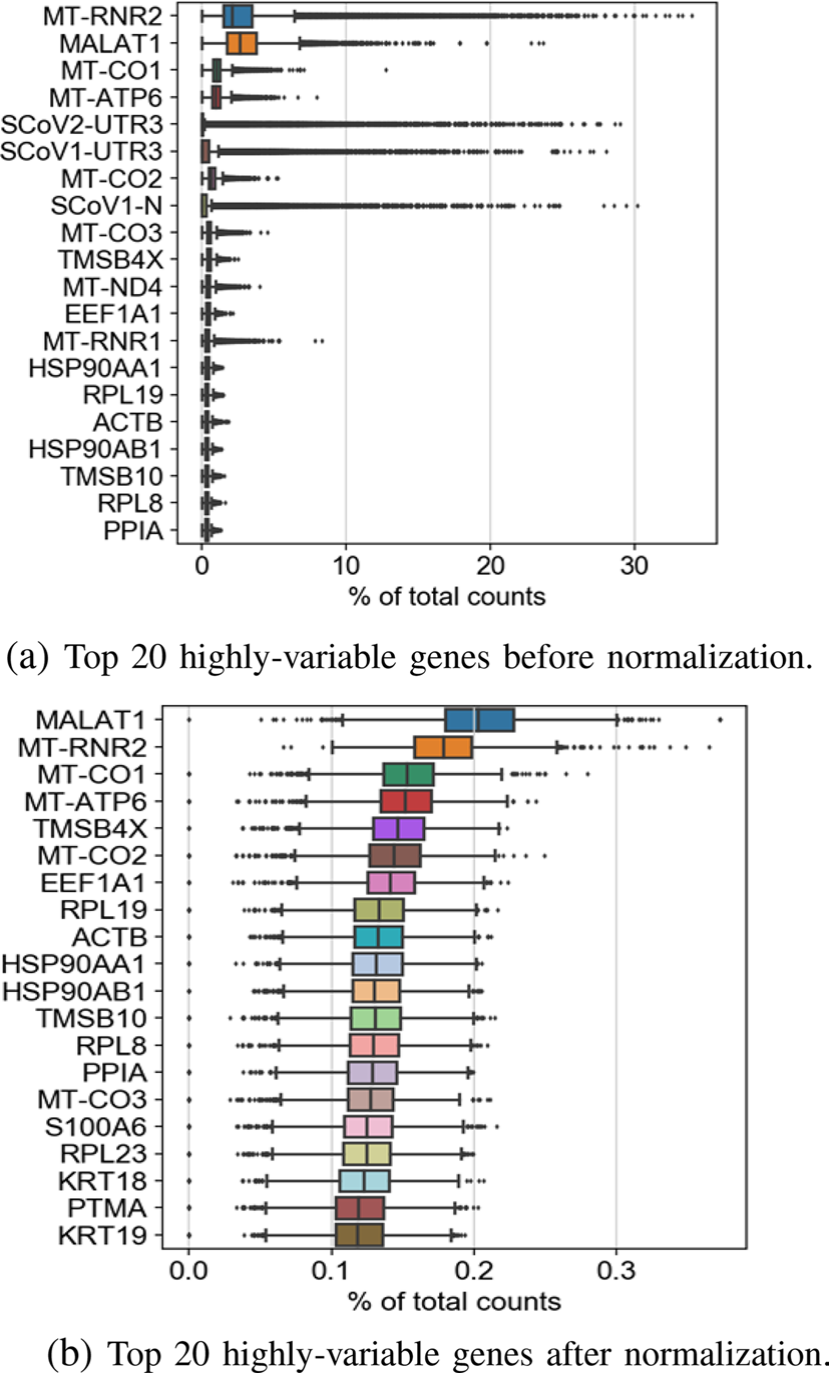
Comparison of the top 20 highly-variable genes before and after normalization.

Visualization of top genes in the dataset is shown in Fig. 4 before and after normalization.

### Dimensionality reduction

The majority of real-life data is multidimensional, and the majority of the high-dimensional data is complex and sparse. Ideally, understanding the data in such dimensions is tricky, and visualization is not possible. Dimensionality reduction is the process of transforming data from a high-dimensional space to a low-dimensional space while retaining some of the original data’s meaningful properties. Working in high-dimensional spaces may be inconvenient for various reasons. For example, data analysis is typically computationally intractable. Single-cell gene expression data is complex and should be well-explored. Each gene is characterized as a dimension in a single-cell expression profile. As such, dimensionality reduction is very productive in summarizing biological attributes in fewer dimensions. Dimensionality reduction is divided into linear and non-linear techniques. We discuss in details in the following subsections.

#### Modified locally linear embedding

Modified LLE is a non-linear dimensionality reduction technique and the enhanced version of LLE. To understand the MLLE, we first need to know the algorithm of LLE. When used for dimensionality reduction, LLE attempts to reveal the manifold underlying structure based on simple geometric intuitions. LLE preserves the data locality in lower dimensions because it reconstructs each sample point from its neighbors. In other words, LLE focuses on finding the lower-dimension representation of high-dimensional data that preserves the locally linear structure of neighboring point patterns most accurately.

In the simplest formulation of LLE, it first identifies *t*-nearest neighbors per data point, as measured by Euclidean distance^24^. One can choose the number of neighbors, *t*, based on rules, metrics, or simply a random number. Consider *n* sample points $\mathbf {X}=\{\mathbf {x}_1,\mathbf {x}_2, \ldots ,\mathbf {x}_n\}$ in high dimensional space of $R^d$. For each sample point $x_i$ and its neighborhood set $N_i = \{\mathbf {x}_t,t \in t_i\}$, one can form *t*-NN graph to construct locally linear structure at that point using the combination of reconstruction weights, $\mathbf {W=}\{w_{ti},t \in t_i\}$, $i= {1, \ldots , n}$. On the basis of this, each data point viewed as a small linear patch of the sub-manifold.

To compute the weights $w_{ti}$ for linear reconstruction of each point, we minimized the cost function with respect to two constraints: (1) each data point $\mathbf {x_i}$ is reconstructed only from its neighbors imposing $w_{ti}=0$ if $\mathbf {x_i}$ does not belong to that set; (2) sum of the weights matrix rows is equal to one, that is $\sum {w_{ti}}=1$. Optimal weights are calculated by solving Eq. (2), the constrained least squares problem:

2 $$\begin{aligned} \min \ \Vert {\mathbf {x}_i - \sum _{t\in t_i}w_{ti}\mathbf {x}_t} \Vert \quad \text {s.t.} \quad \sum _{t \in t_i}w_{ti}=1. \end{aligned}$$

Matrix $\mathbf {G_i}=[..., x_t - x_i,...]_t \in t_i$ can help to formulate the local weight vector $w_{ti}$; Hence, (2) can be reformulated as:

3 $$\begin{aligned} \min \ \Vert {\mathbf {G}_i w} \Vert , \quad \text {s.t.} \quad \sum _{t \in t_i}w_{ti}=1, \quad \text {and} \quad w^T 1_{ti}=1, \end{aligned}$$

where $1_{ti}$ shows the vector of all 1’s with $t_i$-dimension.

Using this formulation, the embedding space *Y* can be computed by singular value decomposition of $G_i$, with *Y* as a solution to the linear combination of $G_i^T {G_i}Y = 1_{ti}$.

Finally, using the same weights computed in the input space, each high-dimensional input sample $\mathbf {x_i}$ is mapped to a lower dimensional point set $\mathbf {Y}=\{\mathbf {y}_1,\mathbf {y}_2, \ldots ,\mathbf {y}_n\}$ in $R^m$ ($m<<d$), representing the manifold’s global internal coordinates.

Equation (4) reflect the locality preservation property by solving a minimization problem over the output manifold.

4 $$\begin{aligned} \min _{Y=\left[ y_1, \ldots ,y_n\right] } \sum _{i=1}^n \Vert {\mathbf {y}_i - \sum _{t\in t_i}w_{ti}\mathbf {y}_t}\Vert ^2 \quad \text {s.t.}\quad YY^T=I. \end{aligned}$$

Regularization is a well-known problem in LLE, which manifests itself in the embedding that distorts the underlying geometry of the manifold. Standard LLE uses an arbitrary regularization parameter concerning the weight matrix’s local trace^25^. MLLE overcomes this problem using multiple weight vectors in each neighborhood, discovering a more stable and enhanced embedding space. MLLE modifies or adjusts the reconstruction weights, which modifies the embedding cost function as follows:

5 $$\begin{aligned} \min _{Y=\left[ y_1, \ldots ,y_n\right] } \sum _{i=1}^n \sum _{l=1}^{s_i} \Vert {\mathbf {y}_i - \sum _{t\in t_i} {w}_{ti}^{l} \mathbf {y}_t}\Vert ^2 \quad \text {s.t.}\quad YY^T=I, \end{aligned}$$

where, $s_i$ is the smallest right singular values of $\mathbf {G_i}$.

This aims to take advantage of the dense relations that exist in the embedding space^26^.

#### Independent component analysis

ICA is an independent and linear dimensionality reduction method. By using simple statistical properties assumptions, ICA learns an efficient linear transformation of the data and attempts to find the underlying structures are presented in the data^27^. Based on the definitions given in^28^, ICA is considered as a special case of projection pursuit; it is a technique for finding relevant projections of multi-dimensional data. Such projections can then be used for enhanced visualization of the clustered data. When ICA is used for visualizing the data, dimensionality reduction becomes its secondary objective^28^. Unlike other approaches, the transformation’s underlying vectors are presumed to be independent of one another. It employs a non-Gaussian data structure, which is crucial for retrieving the transformed underlying data components. ICA aims to find projections of the data that provides estimations of the independent components^28^. When dealing with noise-free data, if there are no assumptions made on the data, ICA can be considered as a high-performance method of exploratory data analysis^28^. Consider $\mathbf {r}$ being a random vector whose elements are $\{\mathbf {r}_1,\mathbf {r}_2, \ldots ,\mathbf {r}_n\}$, and similarly, random vector $\mathbf {s}$ with its elements $\{\mathbf {s}_1,\mathbf {s}_2, \ldots ,\mathbf {s}_n\}$, and also $\mathbf {A}$ is the matrix with elements $a_{ij}$. ICA is a generative model, which captures how the observed data are generated by mixing the components $s_i$ (Eq. 6). The independent components are latent variables, $\mathbf {B}$, which means they are unknown. Also, the mixing matrix ($\mathbf {A}$) is assumed to be unknown and $\mathbf {V}$ is the observed matrix.

6 $$\begin{aligned} \begin{aligned} \mathbf {r}=\mathbf {A}\mathbf {s} \\ \mathbf {V}=\mathbf {A}\mathbf {B}. \end{aligned} \end{aligned}$$

The rows of these matrices are orthogonal to each other. As such, it leads to more independent components than PCA. ICA requires knowing the structure of the data, which is hidden while being analyzed, to untangle their complex relationships and translate them into meaningful measurements. Thus, this feature of ICA is referred to as blind source separation^29^.

#### Other dimensionality reduction methods

We used other dimensionality reduction techniques to compare our proposed method such as Standard LLE, Isomap, Laplacian eigenmap, PCA, and t-SNE. Isomap stands for isometric mapping. Isomap is a non-linear dimensionality reduction method based on the spectral theory that aims to preserve the lower dimension’s geodesic distances. Isomap starts by creating a neighborhood network. After that, it uses graph distance to estimate the geodesic distance among all pairs of points. The eigenvalue decomposition of the geodesic distance matrix finds the lower-dimensional embedding of the data^30^. Laplacian eigenmap is another non-linear technique. It is computationally efficient and maps nearby input patterns to nearby outputs by computing the lower-dimensional representation of a high-dimensional data set. It focuses on preserving local proximity relations among input data points^31^. *t*-SNE is also a non-linear dimensionality reduction technique that is commonly-used for visualization, and has extensively applied in genomic data analysis, and speech processing^6^. On the other hand, PCA is a popular linear technique used for feature extraction or dimensionality reduction. Given a dataset composed of *d*-dimensional points, PCA maps the data linearly to find a subspace in lower-dimensional space so that the dispersion of the data is maximized. It does so via eigen decomposition of the covariance matrix. The principal components (eigenvectors that correspond to the largest eigenvalues) are used to recreate a substantial portion of the original data’s variance^30^.

### Clustering

Performing clustering is one of the critical tasks in single-cell data analysis. Clusters are formed by grouping cells based on their similarity of the gene expression profiles. Distance functions are used to describe expression profile similarity, which employs dimensionality-reduced representations as input. We used the popular clustering technique, *k*-means, an iterative clustering algorithm that groups the data into *C* separate groups of $\mathbf {C}=\{\mathbf {c}_1,\mathbf {c}_2,\ldots ,\mathbf {c}_k\}$ by minimizing the within-cluster dispersion while maximizing the inter-cluster distances. The number of clusters to be formed from the data needs to be specified as an input parameter to the algorithm.

$\textit{k}$-means works in three key steps. The first step is to choose the initial centroids, and the simplest method is to choose $\textit{k}$ samples from the dataset $\mathbf {X}=\{\mathbf {x}_1,\mathbf {x}_2, \ldots ,\mathbf {x}_n\}$. Then, each point in the dataset is allocated to its nearest centroid. The next step involves taking the mean value of all samples allocated to each centroid to update centroids. The algorithm calculates the difference between the old and new centroids, then repeats the last two steps until that value falls below a certain threshold. In other words, it keeps iterating until almost no change in the centroids is observed. The points in the data tends mostly to the centroid which leads to a high degree of cluster compactness or a minimum sum of squared error (*SSE*), as shown in Eq. (7); wherein *n* is the number of samples in the data, $C_j$ is the *j*th cluster, $\mu$ is the mean of the samples, and *x* is the corresponding sample. How to choose the best number of clusters is explained in the next subsection, Parameter Optimization.

7 $$\begin{aligned} SSE= \sum _{i=1}^k \min _{\mu \in C_j} \left( |\mathbf {x}_i - \mathbf {x}_j|\right) ^2. \end{aligned}$$

### Parameter optimization

When applying MLLE, a neighborhood graph, *t*-NN, is created by connecting points that are close to each other. Different measures are used for this purpose, including the number of neighbors, distance from each point to its neighbors, and others. A common measure to determine the sparsity of the neighbor graph is the tolerance factor, which makes the graph sparser or denser. In this regard, we tested different tolerance values on each dataset and selected those values that yielded the best validity index scores, as explained in the Performance Evaluation subsection. With the aim of preserving locality, the number of nearest neighbors, *t*, is a crucial parameter to construct the neighborhood graph. Another critical parameter is the number of clusters, *k*, in the clustering algorithm. The number of nearest neighbors, *t*, are examined within the range [8,24], and the number of clusters *k* for each value of *t* is also assessed, where *k* ranges from 4 to 14, and the validity of indices are calculated for each cluster.

We followed “elbow” method to select the best combination of *t* and *k* with the highest score, considering all the three validity indices, as explained in the Performance Evaluation subsection. By plotting all scores with different number of clusters on the *x*-axis, an elbow-shape point is observed on the plot at a certain value of *k*. A decreasing trend can be observed on the scores, while *k* increases. At this point, which is the best number of clusters, the plot will change rapidly and the trend will lean towards a line almost parallel to the *x*-axis. The corresponding plots are provided in the Supplementary Fig. S1.

### Performance evaluation

Generally speaking, the best clustering is the one that maintains high intra-cluster distance and gives the most compact clusters. In this work, we used the Silhouette coefficient^32^, an evaluation metric that measures either the mean distance between a sample point and all other points in the same cluster or all other points in the next nearest neighbor cluster. Consider a set of clusters $\mathbf {C}$ = $\left\{ \mathbf {C}_1, \mathbf {C}_2,\ldots ,\mathbf {C}_k \right\}$ as the output by a clustering algorithm, $\textit{k}$-means in our case. The Silhouette coefficient, *SH*, for the $i^{th}$ sample point in cluster $\mathbf {C}_j$, where $j = 1, \ldots , k$, can be defined as follows:

8 $$\begin{aligned} \begin{aligned} SH\left( \mathbf {x}_i\right) =\frac{b_c\left( \mathbf {x}_i\right) -w_c\left( \mathbf {x}_i\right) }{max\left( w_c\left( \mathbf {x}_i\right) ,b_c\left( \mathbf {x}_i\right) \right) }, \end{aligned} \end{aligned}$$

where $w_c$ is the mean distance between point $\mathbf {x}_i$ and all other points inside the cluster, within-cluster distance, and $b_c$ is the minimum mean value of the distance between a sample point $\mathbf {x}_i$ and the nearest neighbor cluster, between-cluster distance, which are calculated as:

9 $$\begin{aligned} \begin{aligned} a\left( \mathbf {x}_i\right) =\frac{1}{|\mathbf {C}_k|-1}\sum _{\mathbf {x}_j\in \mathbf {C}_k,i\ne j}d\left( \mathbf {x}_i,\mathbf {x}_j\right) \\ b\left( \mathbf {x}_i\right) =\min _{k\ne i}\frac{1}{|\mathbf {C}_k|}\sum _{j=1}^k d\left( \mathbf {x}_i,\mathbf {x}_j\right) . \end{aligned} \end{aligned}$$

We also used Calinski–Harabasz (CH) and Davies–Bouldin (DB) validity of indices to assess the clustering performance. The CH score is used to evaluate the model, where a higher score tells better-defined clusters^33^. CH is the ratio of the sum of between-cluster and within-cluster dispersion for all clusters, calculated as follows:

10 $$\begin{aligned} CH=\frac{tr\left( \mathbf {S}_B\right) }{tr\left( \mathbf {S}_W\right) } \times \frac{n - k}{k - 1}, \end{aligned}$$

where *n* is size of input samples, $tr(\mathbf {S}_B)$ is the trace of the between-group dispersion matrix and $tr(\mathbf {S}_W)$ is the within-cluster dispersion.

The Davies–Bouldin (DB) index^34^ is another validity measure that is defined as the average of the similarity measure of each cluster. DB is computed as follows:

11 $$\begin{aligned} DB=\frac{1}{k}\sum _{i=1}^k max_{i\ne j}s_{ij}, \end{aligned}$$

where $s_{ij}$ is the ratio between within-cluster distances and between cluster distances, and is calculated as $s_{ij}=\frac{w_i+w_j}{d_{ij}}$. The smaller the DB value is, the better the clustering, and as such, we aim to minimize Eq. (11). Here, $d_{ij}$ is the Euclidean distance between cluster centroids $\mu _i$ and $\mu _j$; and $w_i$ is the within-cluster distance of cluster $\mathbf {C}_k$.

Overall, we used the Silhouette score to evaluate the clustering performance, whereas CH and DB indices were used to verify and find the optimal parameters, namely the best number of clusters for our experiments.

### Cluster annotation

To validate the obtained clusters, we first identified the top 20 differentially expressed genes in each cluster based on the Wilcoxon test and considered them as marker genes that drive high separation among clusters. Marker genes are up or down-regulated in different individual cells, pathways, or GO terms. We used Gene Set Enrichment Analysis, GSEA^35,36^, to annotate the clusters with the corresponding cell types of each group of marker genes. GSEA^37^ is a computational tool that determines whether a predefined set of genes shows a statistically significant level of expression in a specific cell type, biological process, cellular component, molecular function, or biological pathway. The GSEA uses MSigDB, the Molecular Signature Database, to provide gene sets for the gene set enrichment analysis. Also, we employed ToppCluster^38^for Gene Ontology (GO) analysis. ToppCluster is a multi-gene list functional enrichment analysis online tool to identify the GO terms and pathways associated with the top gene lists extracted from each cluster. Pathways were extracted from the MSigDB C2 BIOCARTA (V7.3) database^39^. The corresponding networks are visualized using Cytoscape^40^. We decreased the minimum number of genes present in the corresponding annotations to achieve a better visualization.

## Results and discussion

We first applied the Scanpy pipeline, including its clustering method (Leiden clustering), on the PBMC dataset. The corresponding results are presented in the Supplementary Fig. S3. Then, in order to obtain the most suitable clustering and dimensionality reduction method with higher performance, we employ Scanpy only for the pre-processing step. Since different tools use the same pre-processing approach, it facilitates new innovations emerging in scRNA-seq analysis.

We developed a well-constructed pipeline that can be applied to scRNA-seq data to discover individual cell types. Considering dimensionality reduction and clustering as two significant steps in the pipeline, we explored many ways of untangling the data in two and three dimensions. We found optimal parameters for both dimensionality reduction and clustering that achieve the meaningful separation of cell types and compact clusters. To demonstrate the applicability of our pipeline, we tested it on thirteen datasets of different sizes. Finally, we evaluated our method in terms of both computational and biological perspectives. As *k*-means and, generally, all distance-based methods are known to not work well with non-linear methods such as *t*-SNE, we employed DBSCAN clustering to investigate the behavior in the datasets used in this work. The resulting Silhouette score for the Calu3 dataset is 0.871, which is relatively lower than those of *k*-means whose score is 0.924 for the same datasets. These results along with further discussions are provided in the Supplementary Fig. S2.

### Clustering and cell type discovery

To achieve optimized results, we experimented with all possible combinations of parameters as discussed in the Material and Methods section. As a result, the best parameters that we could obtain for each dataset are depicted in Table 2. In a few datasets, to achieve the best clustering score in the proposed approach, the data is reduced to lower dimensions, such as 5, 6, and 7. Afterward, the data is reduced to three dimensions to visualize and obtain better results. The results of *k*-means clustering combined with each dimensionality reduction method using the best parameters are listed in Table 3. The last column shows the result after applying ICA on the result of clustering combined with MLLE. The clustering scores range from 0 to 1. A score close to 1 represents good quality clustering, with 1 being the best, while a score near zero indicates that the clusters are not well defined.

**Table 2:** Parameters used for experiments; *t* is the number of neighbors and *K* is the number of clusters. These parameters are generated considering both dimensionality reduction and clustering together.

Dataset	*t*	No. of dimensions	Tolerance	*k*
Baron_human1	10	6	1e−12	14
	23	3	1e−10	
Baron_human2	8	3	1e−12	14
Baron_human3	16	7	1e−12	14
	8	3	1e−8	
Baron_human4	9	6	1e−12	14
	22	3	1e−12	
Baron_mouse1	17	3	1e−12	13
Baron_mouse2	11	6	1e−12	13
	20	3	1e−8	
Muraro	10	5	1e−3	6
	11	3	1e−7	
Segerstolpe	10	5	1e−3	6
	9	3	1e−8	
Xin	15	6	1e−12	6
	25	3	1e−3	
Wang	8	3	1e−12	6
H1299 scRNA-seq	11	3	1e−8	7
Calu3 scRNA-seq	12	7	1e−3	7
	11	3	1e−5	
PBMC	8	5	1e−12	8
	25	3	1e−12

**Table 3:** Silhoutte scores comparison of proposed method with other dimensionality reduction techniques.

Dataset name	t-SNE	PCA	Isomap	Standard LLE	Eigenmaps	MLLE	MLLE+ICA
Baron_human1	0.244	0.364	0.498	0.524	0.839	**0.908**	0.904
Baron_human2	0.231	0.428	0.543	0.614	0.823	**0.906**	0.905
Baron_human3	0.243	0.377	0.522	0.467	0.826	**0.990**	0.976
Baron_human4	0.239	0.424	0.614	0.538	0.896	0.910	**0.912**
Baron_mouse1	0.231	0.400	0.422	0.448	0.472	0.881	**0.917**
Baron_mouse2	0.221	0.414	0.530	0.684	0.779	0.941	**0.943**
Muraro	0.258	0.494	0.532	0.738	0.913	0.933	**0.944**
Segerstolpe	0.231	0.410	0.399	0.400	0.537	**0.960**	0.956
Xin	0.242	0.445	0.481	0.494	0.751	**0.899**	0.888
Wang	0.230	0.484	0.442	0.745	0.608	0.993	**0.996**
H1299 scRNA-seq	0.245	0.269	0.701	0.683	0.782	0.938	**0.943**
Calu3 scRNA-seq	0.361	0.232	0.494	0.452	0.798	0.889	**0.924**
PBMC	0.244	0.401	0.622	0.621	0.632	0.867	**0.876**

Significant values are in bold.

**Figure 5:**
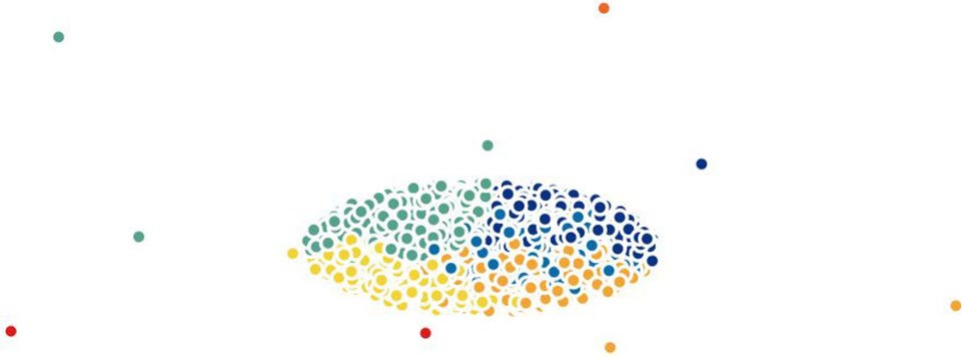
Two-dimensional t-SNE projection of cells colored by *k*-means clustering applied on high-dimensional original data (H1299 scRNA-seq); outliers have been removed to enhance visualization.

**Figure 6:**
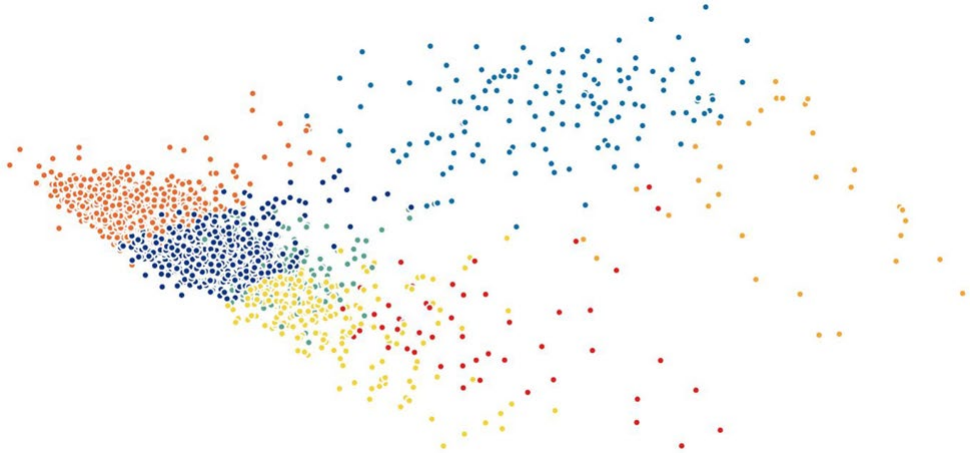
Two-dimensional PCA projection of cells colored by *k*-means clustering applied on high-dimensional original data (H1299 scRNA-seq).

**Figure 7:**
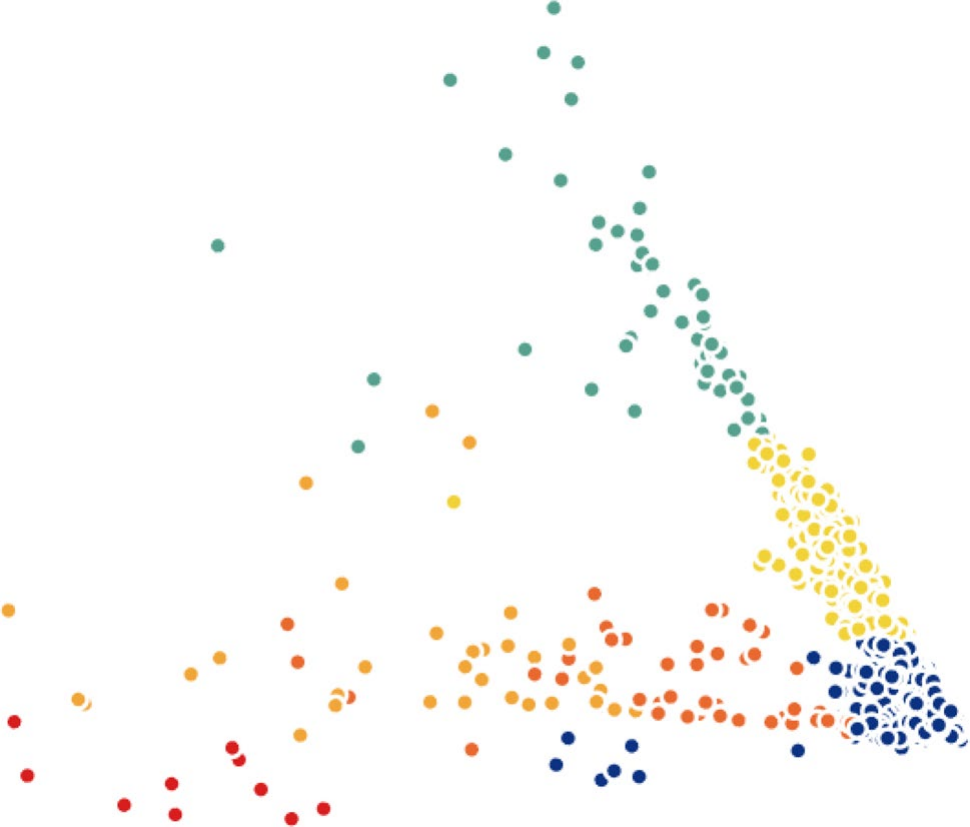
Two-dimensional Laplacian eigenmap projection of cells colored by *k*-means clustering applied on high-dimensional original data (H1299 scRNA-seq); outliers have been removed to enhance visualization.

**Figure 8:**
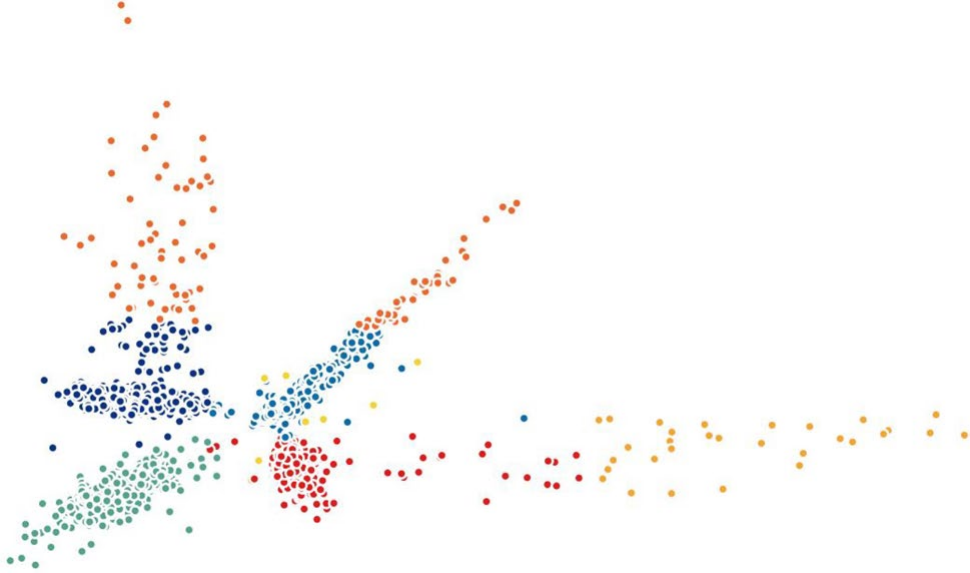
Two-dimensional Isomaps projection of cells colored by *k*-means clustering applied on high-dimensional original data (H1299 scRNA-seq).

**Figure 9:**
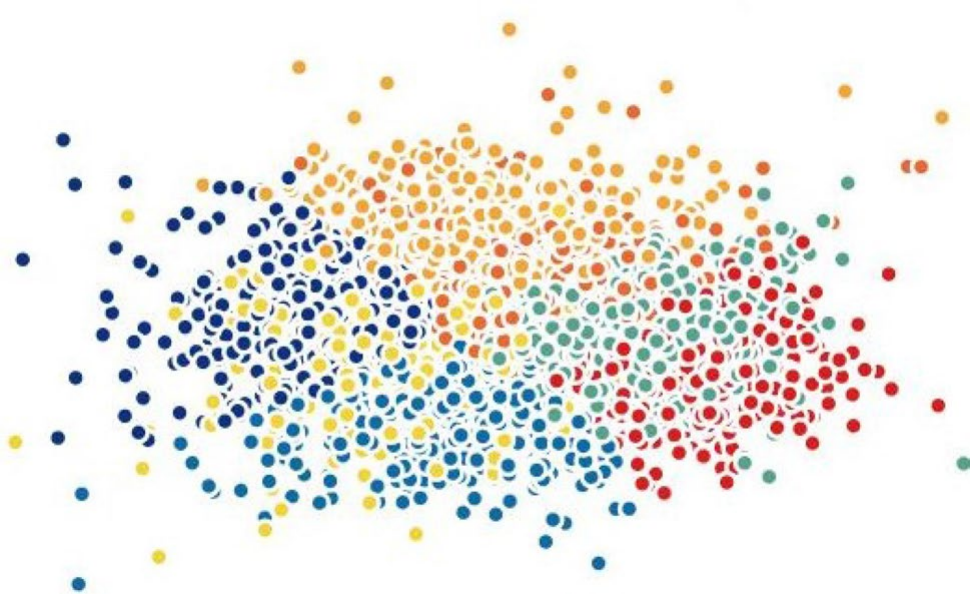
Two-dimensional Standard LLE projection of cells colored by *k*-means clustering applied on high-dimensional original data (H1299 scRNA-seq);outliers have been removed to enhance visualization.

**Figure 10:**
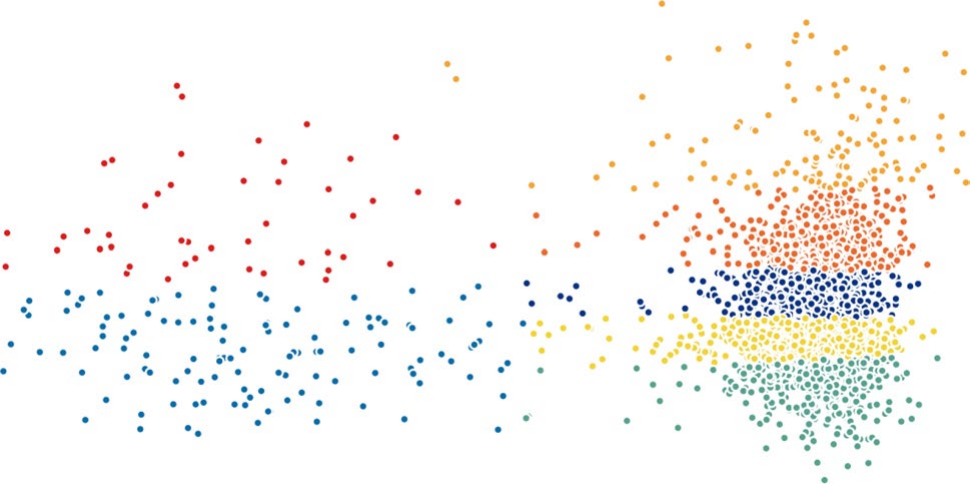
Two-dimensional ICA projection of cells colored by *k*-means clustering applied on high-dimensional original data (H1299 scRNA-seq).

When testing other widely-used techniques such as *t*-SNE and PCA, we noticed that both methods were not efficient in separating the data into well-defined clusters. On the other hand, the results of Isomap and Laplacian Eigenmaps show slightly better performance comparatively. To demonstrate this statement graphically, we visualize the two-dimensional projection of cells resulting from different dimensionality reduction methods and colored by *k*-means clustering on the H1299 scRNA-seq dataset, in Figs. 5, 6, 7, 8, 9 and 10. Moreover, three-dimensional results on the same dataset are presented in Supplementary Fig. S5, Supplementary Material.

**Figure 11:**
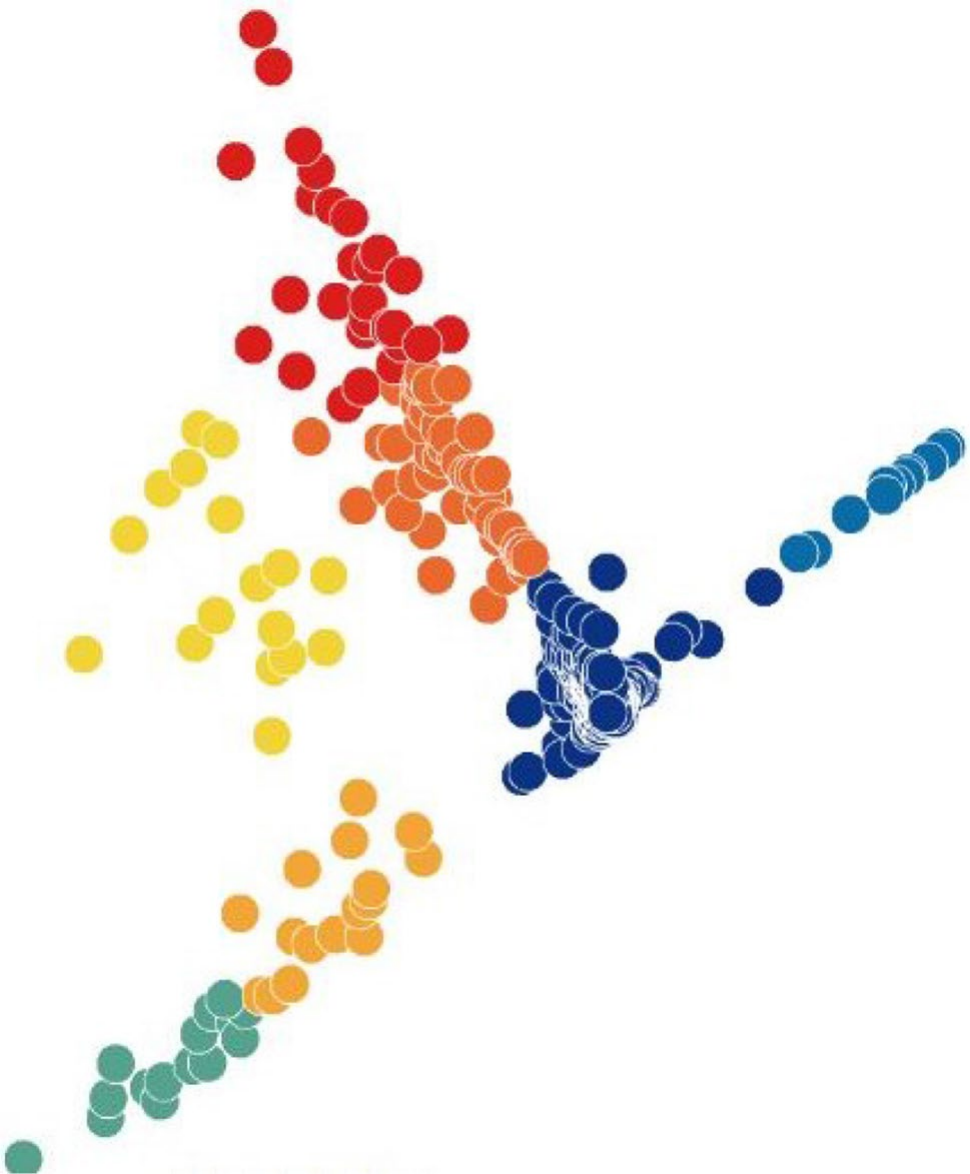
Three-dimensional MLLE projection of cells colored by *k*-means clustering applied on high-dimensional original data (H1299 scRNA-seq).

**Figure 12:**
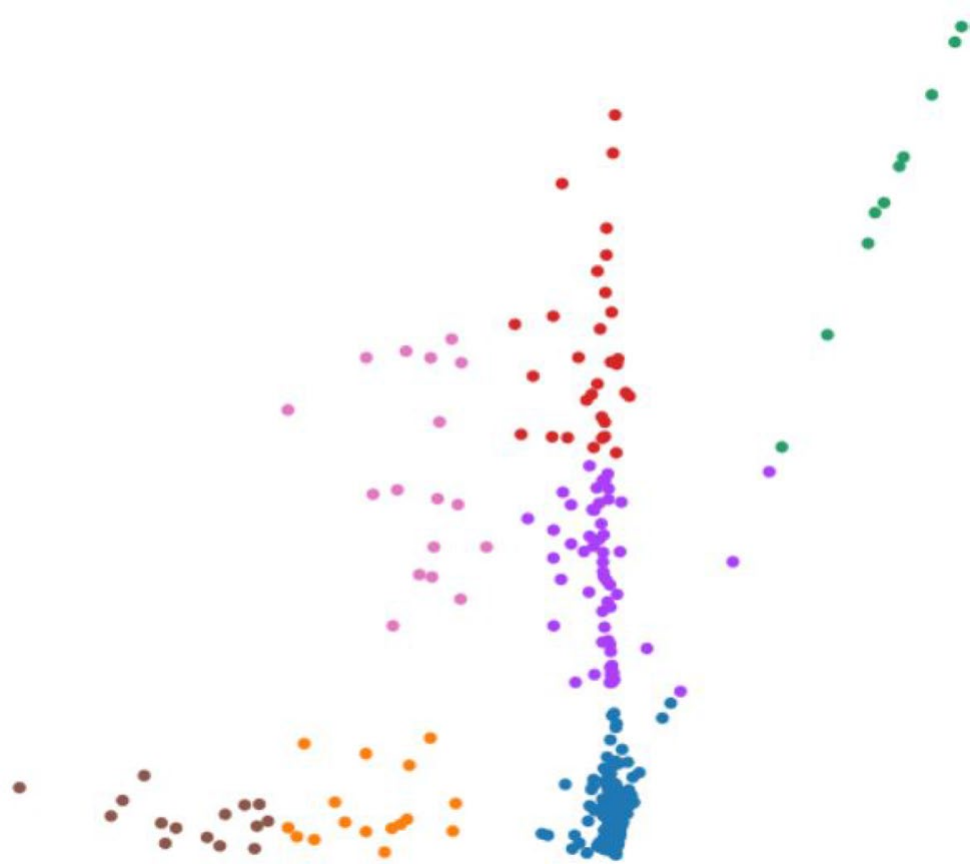
Two-dimensional ICA projection of cells colored by *k*-means clustering applied to the three-dimensional points output by MLLE on the H1299 scRNA-seq dataset.

Finally, we investigated MLLE and found the most insightful cluster separation in most of the datasets. This outcome demonstrates the power of MLLE in exploring the data’s dense and complex relations, creating better embeddings in lower-dimensional spaces. We performed an additional dimensionality reduction step that uses ICA to enhance the visualization of the clusters. The last column of Table 3 shows that MLLE combined with ICA improves the overall results except for some datasets in which we do not notice much difference; very negligible difference of 0.004 (Baron_human1), 0.001 (Baron_human2), 0.014 (Baron_human3), 0.004 (Segerstolpe), and 0.011 (Xin) can ignore them. To achieve a better view of the impact of ICA on the MLLE transformation, we show a visual comparison of the clusters in Figs. 11 and 12. Two-dimensional ICA projection of the cells applied to the three-dimensional MLLE data shows the best visualization and clustering scores (Fig. 12). When applied alone, ICA performs very poorly with significantly inseparable clusters (Fig. 10). This result is because ICA is limited to linear transformations. On the other hand, manifold learning techniques consider data locally. As such, it can reveal complex relationships among the data points in higher-dimensional spaces. We instead applied ICA on the lower-dimensional data because we observed well-marked “lines” or “axes” in the three-dimensional data, which led us to conclude that we could apply ICA to learn the linearly independent components, not necessarily orthogonal. Applying ICA reveals some hidden, complex relationships among the cells in the clusters, which are not noticeable in three dimensions.

### Biological assessment

As shown in Table 4, some of the pancreatic cell types are found for pancreas datasets, such as the Baron human dataset within well-defined gene sets in the C8 collection of MSigDB, which includes cell type signature’s gene sets. They include ’MURARO PANCREAS ALPHA CELL’, ’MURARO PANCREAS ENDOTHELIAL CELL’, ’MURARO PANCREAS MESENCHYMAL STROMAL CELL’, ’MURARO PANCREAS DUCTAL CELL’, and ’MURARO PANCREAS ACINAR CELL’.Other cell types such as HB2 is a cell line originated by epithelial cells.

**Table 4:** Identified cell types for Baron_human1 dataset.

Cell types	Cluster number
Alpha	0
CD34	1
Mesenchyme stem cells	2
Jurkat cells (T lymphocyte)	3
Endothelial	4
Mesenchyme stromal cells	5
Ductal	6
Endothelial	7
Acinar	8
Myeloid cells	9
Intestine cells	10
Macrophage	11
HB2 cells	12
T-cells	13

Regarding the PBMC dataset, we identified gene sets from MSigDB based on the ranked gene sets, including TRAVAGLINI LUNG CD8 NAIVE T CELL, TRAVAGLINI LUNG PLATELET MEGAKARYOCYTE CELL, AIZARANI LIVER C18 NK NKT CELLS 5, DURANTE ADULT OLFACTORY NEUROEPITHELIUM DENDRITIC CELLS, TRAVAGLINI LUNG OLR1 CLASSICAL MONOCYTE CELL, FAN OVARY CL12 T LYMPHOCYTE NK CELL 2, and AIZARANI LIVER C34 MHC II POS B CELLS. The corresponding cell types are presented in the Table 5. Moreover, we observed some reported marker genes of the PBMC dataset in some clusters, which are shown in the same table as well.

**Table 5:** Identified cell types for PBMC dataset.

Cell types	Cluster number	Marker genes
CD8 T cells	0	CD8A
Megakaryocytes	1	PPBP
NK cells	2	
Dendritic cells	3	FCER1A, CST3
Classical monocytes	4	
T cells	5	
B cells	6	
CD14+ monocytes	7	CD14

**Figure 13:**
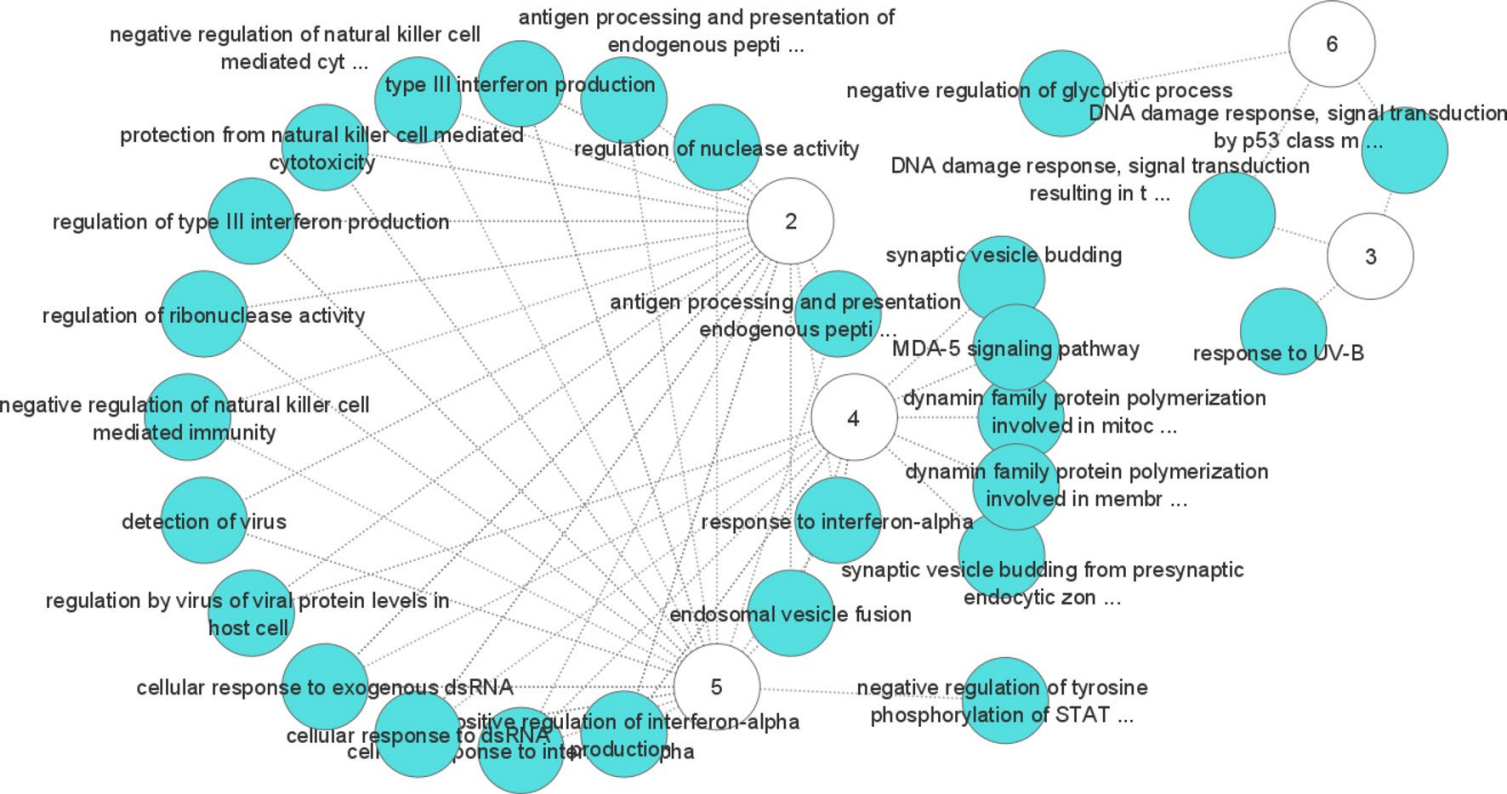
A set of biological process that are enriched by marker genes in H1299 scRNA-seq dataset. The numbers show the clusters and edges shows the link between a cluster and a biological process term.

**Figure 14:**
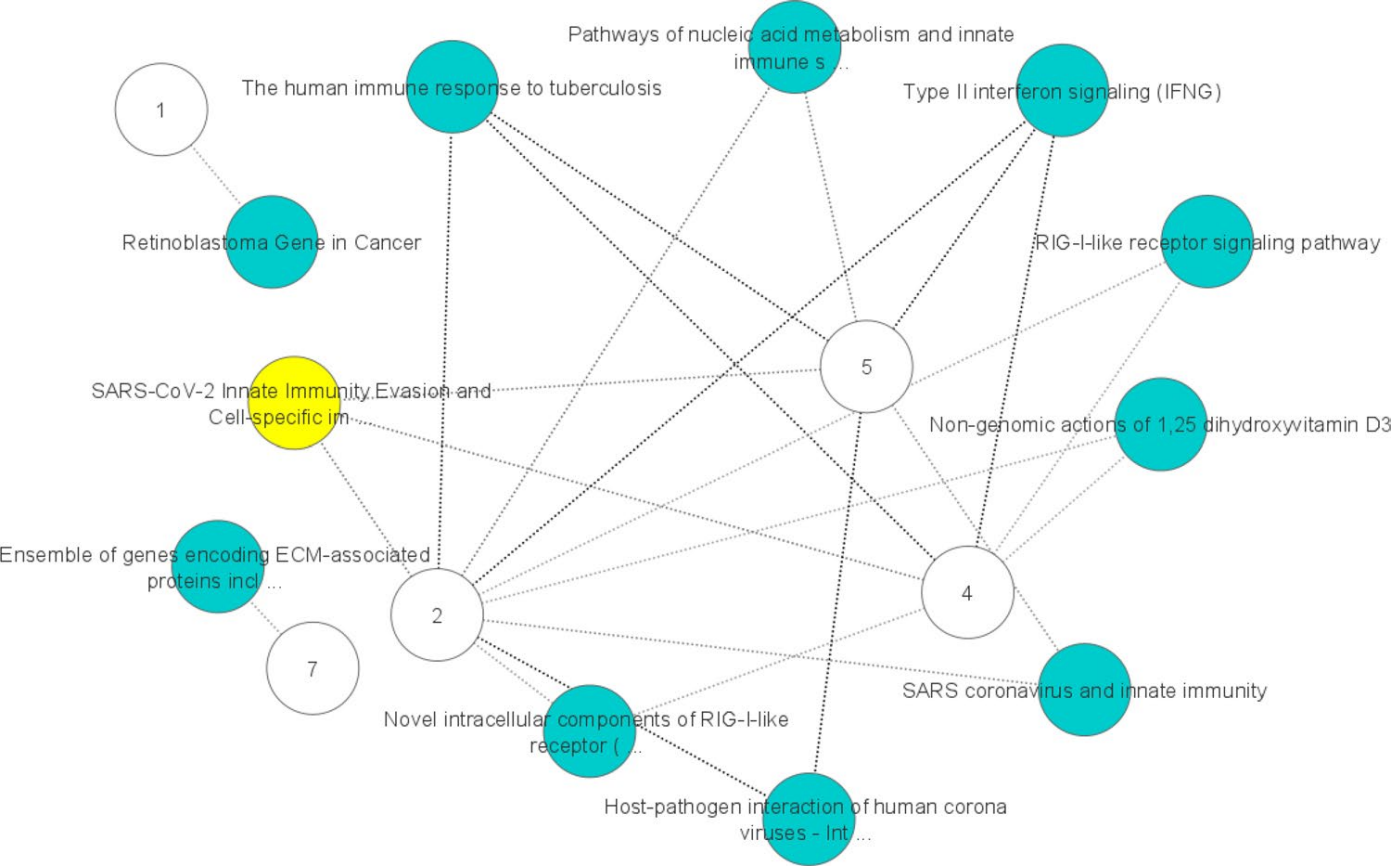
Pathways that are enriched by marker genes in H1299 scRNA-seq dataset. The numbers show the clusters and edges shows the link between a cluster and a pathway. The nodes that are highlighted in yellow show the SARS-CoV-2 cell-specific pathway. Most of the other green nodes reveal the shared and cluster-specific functional pathways in the immune system.

Additionally, the visualization of the networks of GO terms and pathways associated with the corresponding marker genes of the H1299 scRNA-seq dataset are depicted in Figs. 13 and 14, respectively. For each cluster, we identified a set of biological process or pathway terms that connect with a term that is significantly associated with the top 20 gene list in that cluster. By observing Fig. 14, some significant pathways are found to be enriched in immunity functions and signaling, including SARS-CoV-2 innate Immunity Evasion, Host–pathogen interaction of human coronaviruses, SARS coronavirus and innate immunity, Type II interferon signaling (IFNG), and the human immune response to tuberculosis. Also, Fig. 13 shows that most biological processes associated with immunity functions, including response to interferon-alpha, protection from a natural killer cell, type III interferon production, regulation by virus of viral protein levels in a host cell, and detection of virus, among others. In addition, we obtained a list of overlapping marker genes involved in Herpes simplex virus 1 (HSV-1) infection and the Influenza A pathway. These findings suggest potential markers for subsequent medical treatment or drug discovery by comparing similar diseases in terms of functionality. Moreover, although numerous findings suggest potential links between HSV-1 and Alzheimer’s disease, a causal relationship has not been demonstrated yet^41^.

## Conclusion and future work

This work focuses on the identification of different cell types using manifold learning combined with clustering techniques on scRNA-seq data. Identifying similarities that result from structural, functional, or evolutionary relationships among the genes is the primary goal of clustering the cells. Our proposed two-step representation learning approach demonstrated that *k*-means clustering technique combined with Modified LLE leads to improved clustering output and meaningful organization of cell clusters by “untangling” the complex, hidden relationship in a higher-dimensional space.

Non-linear dimensionality reduction methods have been shown to be very powerful as they preserve the locality of the data from higher to lower dimensions. UMAP is one of the most commonly-used non-linear dimensionality reduction technique, and has been shown to perform well on large-scale scRNA-seq data. However, for dimensionality reduction, UMAP is not as efficient as MLLE on high-dimensional cytometry, especially when combined with clustering to enhancing the visualization of the clustering results. This behavior of MLLE has been observed in our experiments. A comparative analysis with UMAP in the Supplementary Material, Supplementary Fig. S4, confirms this observation.

Moreover, performing ICA on transformed data after applying manifold learning techniques provides enhanced view of the data in a reduced space. Evaluating the incidence of ICA as a visualization scheme and further reduction step, after applying MLLE, shows better clustering and enhanced visualization simultaneously. This trend leads to a research avenue that involves a combination of non-linear manifold learning techniques followed by linear methods, which has shown to be more powerful than conventional methods such as PCA or ICA applied alone.

Using multiple benchmark datasets shows the effectiveness of our proposed method. Performing gene set enrichment analysis to annotate a set of HVGs obtained from each cluster reveals biomarker genes involved in different gene ontology terms.

There are some other potential applications for investigating scRNA-seq data, even beyond cell type identification. Using an extension of the proposed method by employing other manifold or deep learning techniques on the other epigenetic challenges in scRNA-seq data analysis, such as trajectory analysis, is our next step.

## Supplementary Information

Supplementary Figures.

## Data Availability

The source code is available in the GitHub repository, at https://github.com/saiteja-danda/Non-linear-and-linear-techniques-for-dimensionality-reduction-visualization-on-single-cell-data.git and is released under MIT license.
