# Supplementary material for: Discovering cell types using manifold learning and enhanced visualization of single-cell RNA-Seq data
Source: Sci Rep. 2022 Jan 7;12:120. doi: 10.1038/s41598-021-03613-0 (PMC8742092; doi:10.1038/s41598-021-03613-0)

Supplementary materials for

Discovering Cell Types Using Manifold Learning and Enhanced Visualization of Single-cell RNA-Seq Data

Akram Vasighizaker, Saiteja Danda, Luis Rueda

Figure S1: *Clustering performance based on Silhouette score (SH), Calinski-Harabasz (CH), and Davies-Bouldin (DB) on H1299 scRNAseq dataset for different number of clusters and nearest neighbors. A higher score of SH and CH tells better-defined clusters, but the smaller DB value show better clustering. The "elbow" method have been used to select the optimal number of clusters by fitting the model with a range of values of K.*

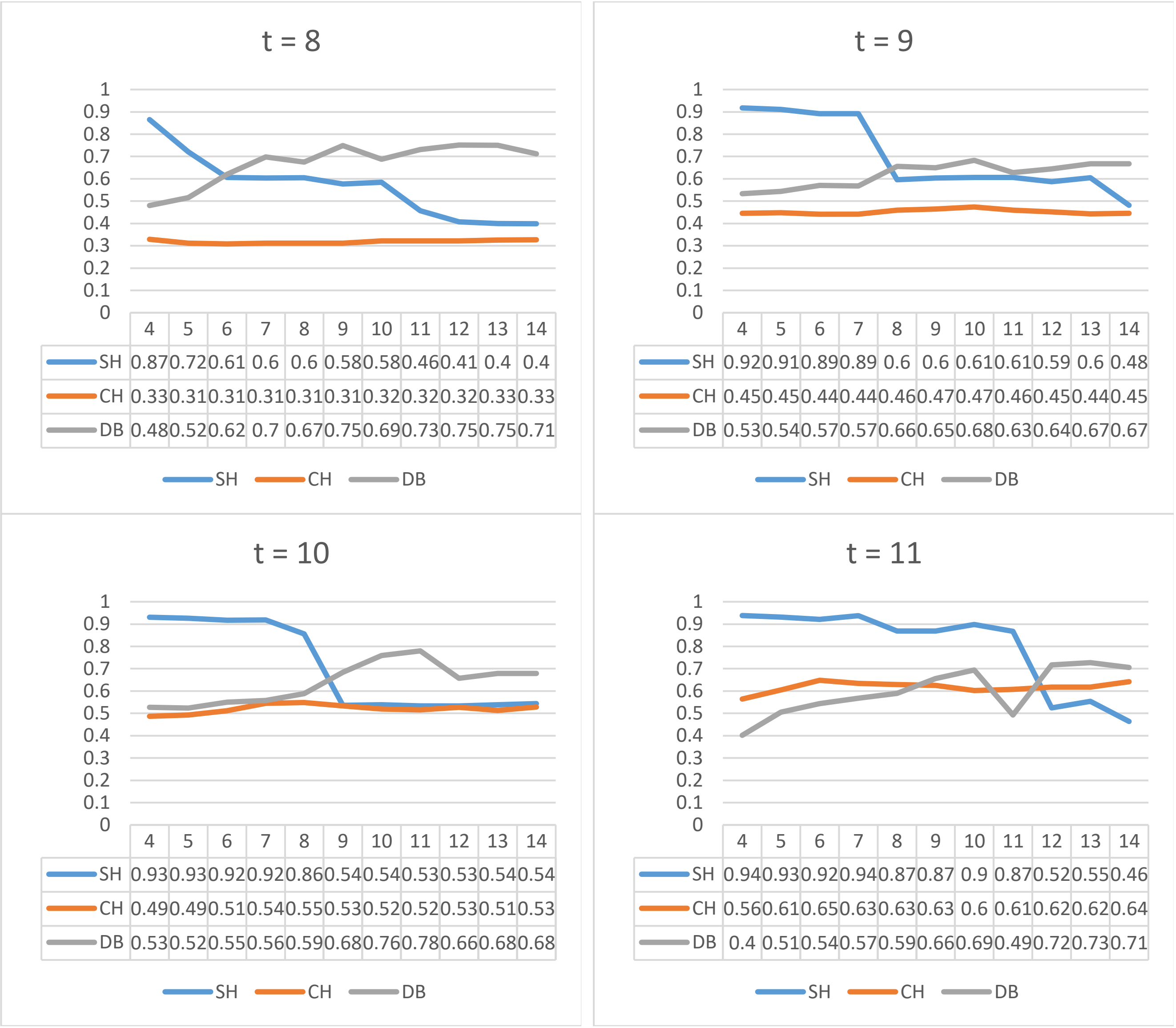

t = 12

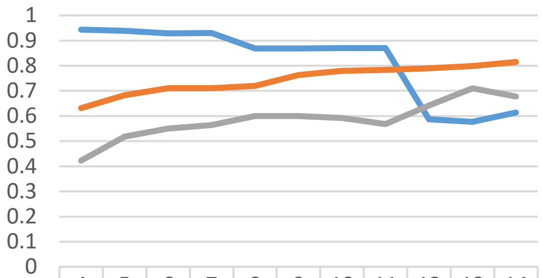

	4	5	6	7	8	9	10	11	12	13	14
SH	0.94	0.94	0.93	0.93	0.87	0.87	0.87	0.87	0.59	0.58	0.61
CH	0.63	0.68	0.71	0.71	0.72	0.76	0.78	0.78	0.79	0.8	0.81
DB	0.42	0.52	0.55	0.56	0.6	0.6	0.59	0.57	0.64	0.71	0.68

SH CH DB

t = 13

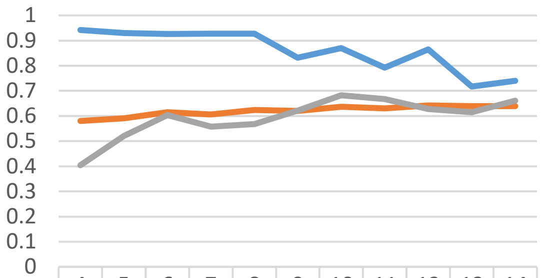

	4	5	6	7	8	9	10	11	12	13	14
SH	0.94	0.93	0.93	0.93	0.93	0.83	0.87	0.79	0.87	0.72	0.74
CH	0.58	0.59	0.61	0.61	0.62	0.62	0.64	0.63	0.64	0.64	0.64
DB	0.4	0.52	0.6	0.56	0.57	0.62	0.68	0.67	0.63	0.61	0.66

SH CH DB

t = 14

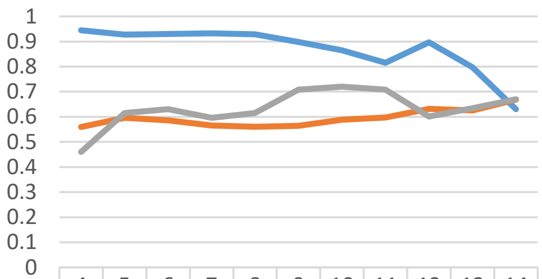

	4	5	6	7	8	9	10	11	12	13	14
SH	0.94	0.93	0.93	0.93	0.93	0.9	0.87	0.81	0.9	0.8	0.63
CH	0.56	0.6	0.59	0.57	0.56	0.56	0.59	0.6	0.63	0.63	0.67
DB	0.46	0.62	0.63	0.6	0.61	0.71	0.72	0.71	0.6	0.63	0.67

SH CH DB

t =15

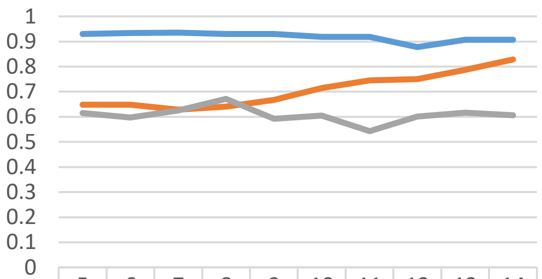

	5	6	7	8	9	10	11	12	13	14
SH	0.93	0.93	0.94	0.93	0.93	0.92	0.92	0.88	0.91	0.91
CH	0.65	0.65	0.63	0.64	0.67	0.71	0.75	0.75	0.79	0.83
DB	0.62	0.6	0.63	0.67	0.59	0.6	0.54	0.6	0.62	0.61

SH CH DB

t =16

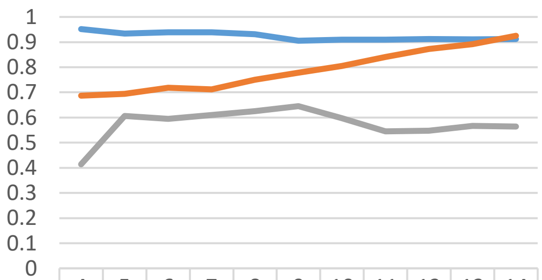

	4	5	6	7	8	9	10	11	12	13	14
SH	0.95	0.93	0.94	0.94	0.93	0.91	0.91	0.91	0.91	0.91	0.91
CH	0.69	0.69	0.72	0.71	0.75	0.78	0.81	0.84	0.87	0.89	0.92
DB	0.41	0.61	0.59	0.61	0.63	0.65	0.6	0.55	0.55	0.57	0.56

SH CH DB

t = 17

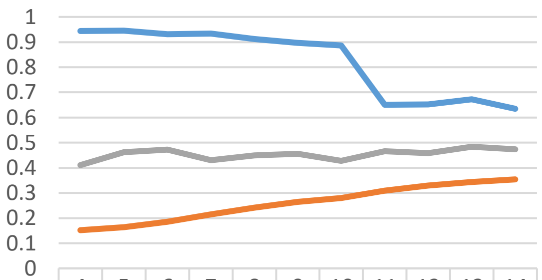

	4	5	6	7	8	9	10	11	12	13	14
SH	0.94	0.95	0.93	0.93	0.91	0.9	0.89	0.65	0.65	0.67	0.63
CH	0.15	0.16	0.18	0.22	0.24	0.26	0.28	0.31	0.33	0.34	0.35
DB	0.41	0.46	0.47	0.43	0.45	0.46	0.43	0.47	0.46	0.48	0.47

SH CH DB

t = 18

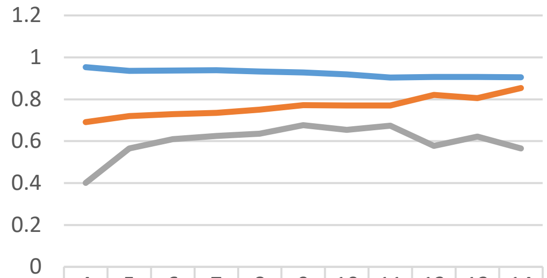

	4	5	6	7	8	9	10	11	12	13	14
SH	0.95	0.94	0.94	0.94	0.93	0.93	0.92	0.9	0.91	0.91	0.91
CH	0.69	0.72	0.73	0.74	0.75	0.77	0.77	0.77	0.82	0.81	0.85
DB	0.4	0.56	0.61	0.62	0.64	0.68	0.65	0.67	0.58	0.62	0.57

SH CH DB

t =19

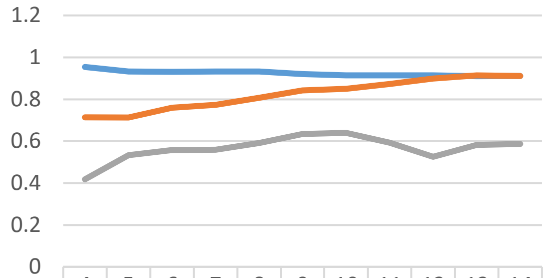

	4	5	6	7	8	9	10	11	12	13	14
SH	0.95	0.93	0.93	0.93	0.93	0.92	0.91	0.91	0.91	0.91	0.91
CH	0.71	0.71	0.76	0.77	0.81	0.84	0.85	0.87	0.9	0.91	0.91
DB	0.42	0.53	0.56	0.56	0.59	0.63	0.64	0.59	0.53	0.58	0.59

SH CH DB

t =20

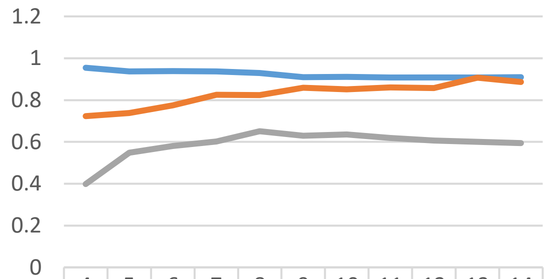

	4	5	6	7	8	9	10	11	12	13	14
SH	0.95	0.94	0.94	0.94	0.93	0.91	0.91	0.91	0.91	0.91	0.91
CH	0.72	0.74	0.77	0.83	0.82	0.86	0.85	0.86	0.86	0.91	0.89
DB	0.4	0.55	0.58	0.6	0.65	0.63	0.64	0.62	0.61	0.6	0.59

SH CH DB

t =21

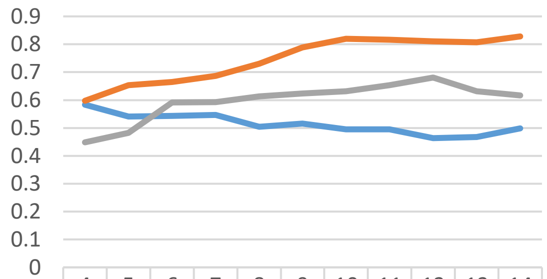

	4	5	6	7	8	9	10	11	12	13	14
SH	0.58	0.54	0.54	0.55	0.5	0.52	0.5	0.49	0.46	0.47	0.5
CH	0.6	0.65	0.67	0.69	0.73	0.79	0.82	0.82	0.81	0.81	0.83
DB	0.45	0.48	0.59	0.59	0.61	0.62	0.63	0.65	0.68	0.63	0.62

SH CH DB

t = 22

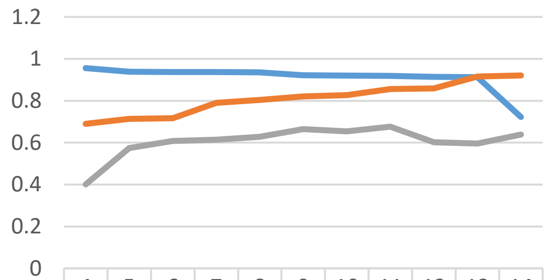

	4	5	6	7	8	9	10	11	12	13	14
SH	0.96	0.94	0.94	0.94	0.93	0.92	0.92	0.92	0.91	0.91	0.72
CH	0.69	0.71	0.72	0.79	0.8	0.82	0.83	0.86	0.86	0.92	0.92
DB	0.4	0.57	0.61	0.61	0.63	0.66	0.65	0.68	0.6	0.6	0.64

SH CH DB

t = 23

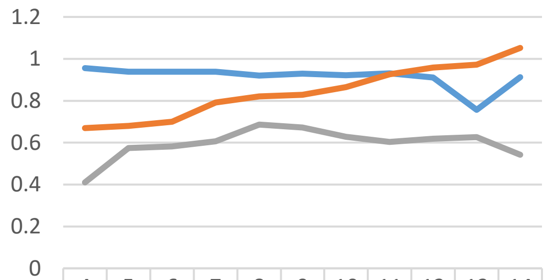

	4	5	6	7	8	9	10	11	12	13	14
SH	0.96	0.94	0.94	0.94	0.92	0.93	0.92	0.93	0.91	0.76	0.91
CH	0.67	0.68	0.7	0.79	0.82	0.83	0.86	0.93	0.96	0.97	1.05
DB	0.41	0.57	0.58	0.61	0.69	0.67	0.63	0.6	0.62	0.63	0.54

SH CH DB

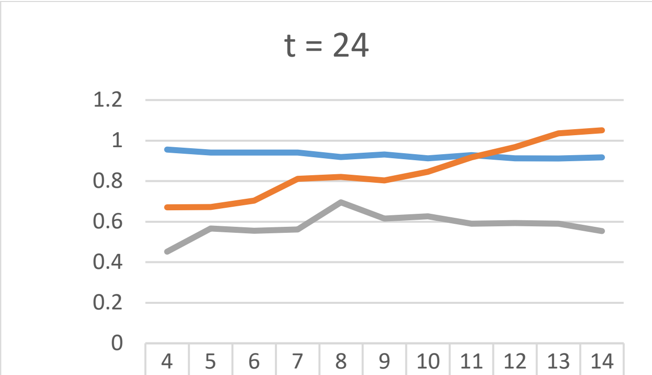

	4	5	6	7	8	9	10	11	12	13	14
SH	0.96	0.94	0.94	0.94	0.92	0.93	0.91	0.93	0.91	0.91	0.92
CH	0.67	0.67	0.7	0.81	0.82	0.8	0.85	0.92	0.97	1.04	1.05
DB	0.45	0.57	0.55	0.56	0.7	0.62	0.63	0.59	0.59	0.59	0.55

SH CH DB

Figure S2: *DBSCAN clustering results on Calu3 dataset; for the purpose of enhanced visualization, outliers have been removed.*

a) *3D MLLE projection of cells colored by DBSCAN clustering applied to the original high-dimensional dataset*

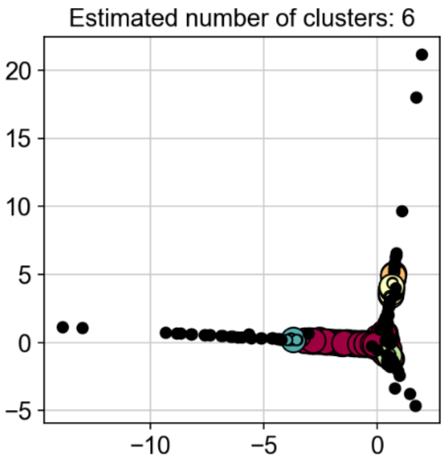

b) *2D ICA projection of cells colored by DBSCAN clustering applied to the 3D output of MLLE; Silhouette score=87.1%*

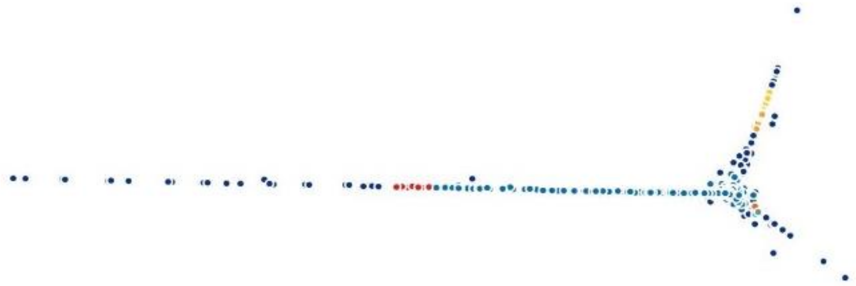

Figure S3: *The result of scanpy pipeline on PBMC dataset using Leiden clustering*

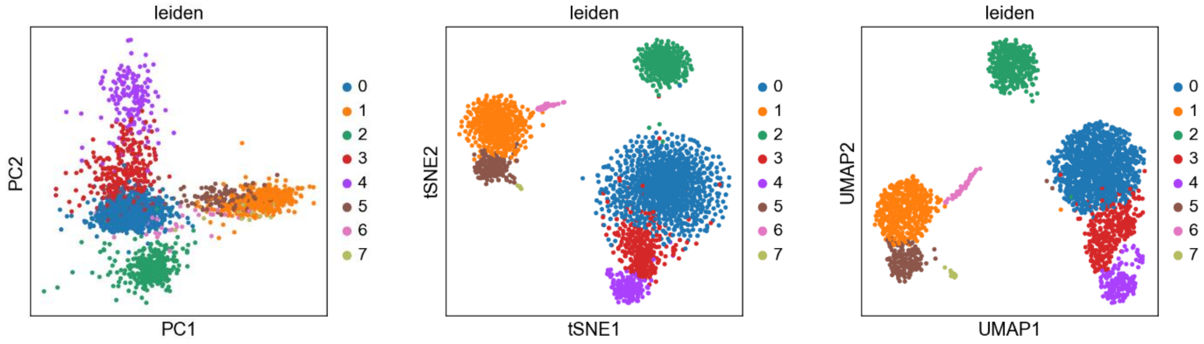

Silouettee score : 0.12 *Silouettee score : 0.42* *Silouettee score : 0.49*

__Figure S4:__ Comparative analysis of employing UMAP and MLLE as non-linear dimensionality reduction techniques combined with k-means clustering on PBMC dataset in the proposed method

a) *k-means clustering on 3D UMAP; Silhouette score= 27.2%*

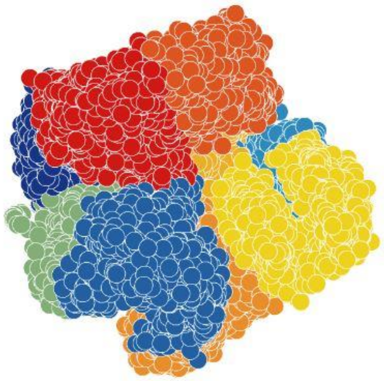

b) *k-means clustering on 3D MLLE; Silhouette score=0.86*

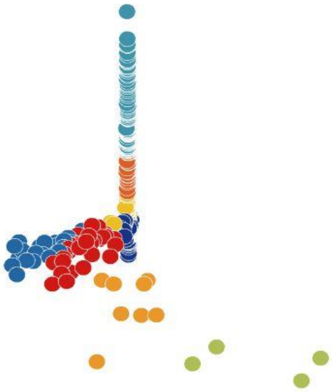

c) *2D ICA + k-means clustering on 3D UMAP; Silhouette score=36.2%*

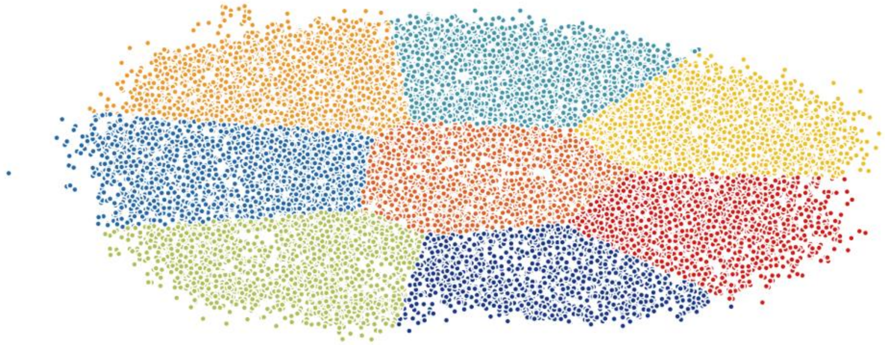

d) *ICA + k-means clustering on 3D MLLE; Silhouette score=87%*

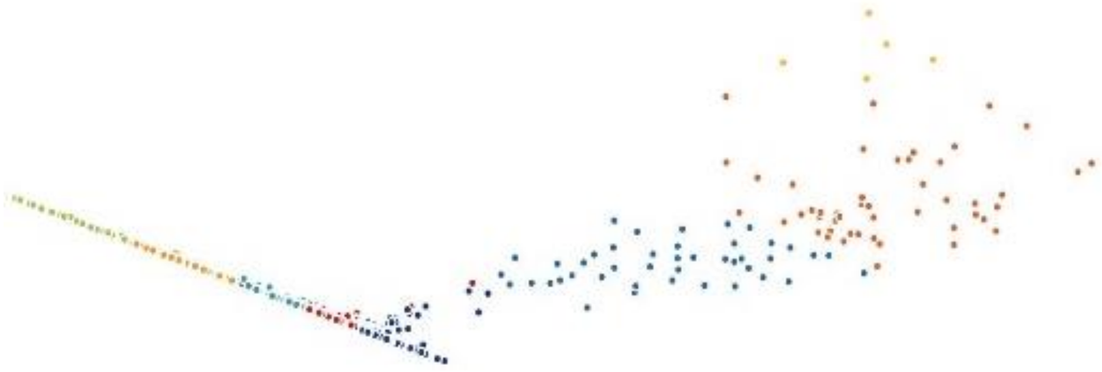

Figure S5: *Three-dimensional projections of cells result from different dimensionality reduction methods and colored by k-means clustering on H1299 scRNA-seq dataset*

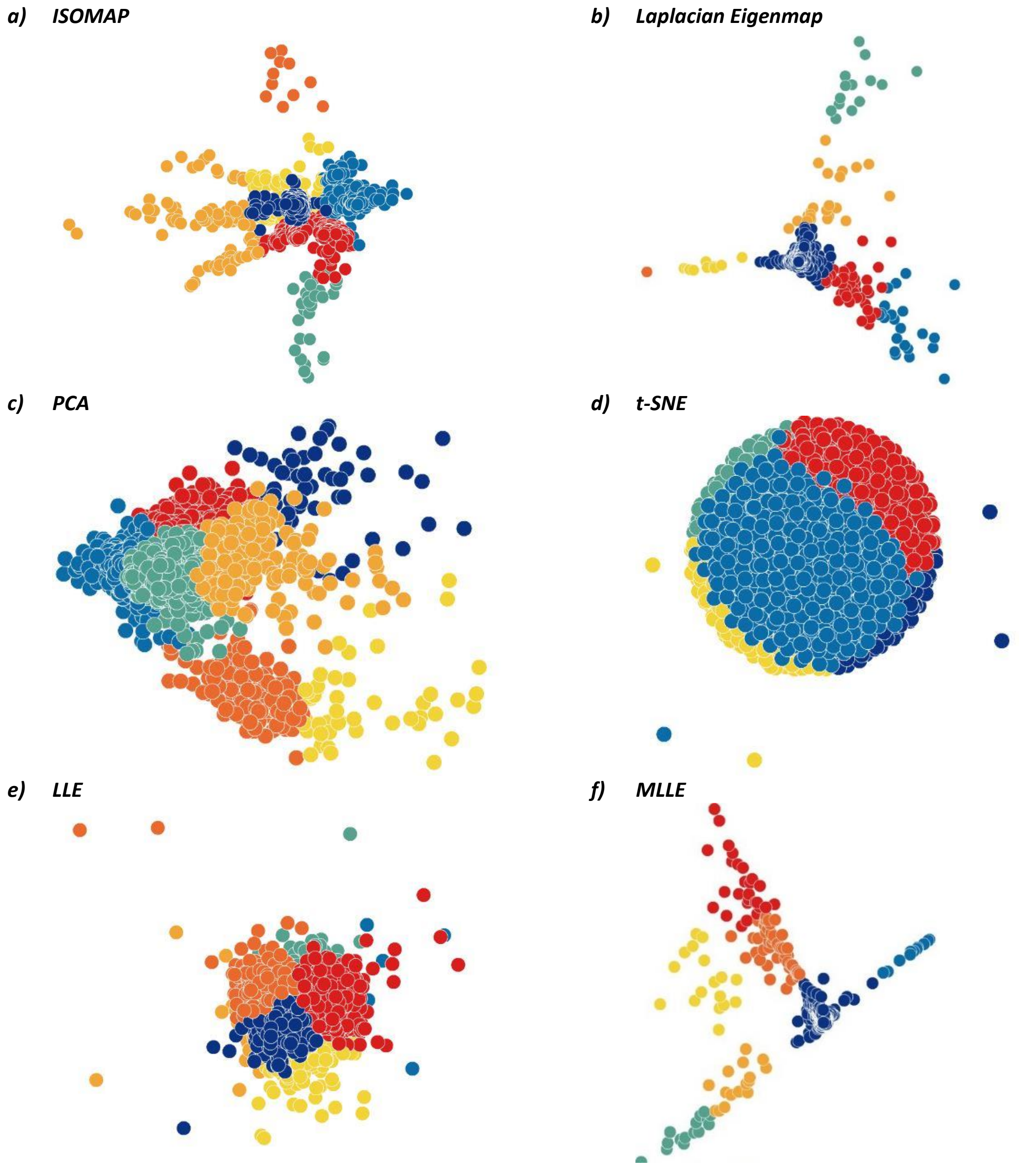

Supplement: Supplementary file 1 — Supplementary Figures. [file 41598_2021_3613_MOESM1_ESM.pdf]
